# Breakfast skipping and its relationship with academic performance in Ethiopian school-aged children, 2019

**DOI:** 10.1186/s40795-022-00545-4

**Published:** 2022-06-01

**Authors:** Lulu Abebe, Nebiyu Mengistu, Tinsae Shemelise Tesfaye, Robel Hussen Kabthymer, Wondwosen Molla, Desalegn Tarekegn, Aregahegn Wudneh, Misrak Negash Shonor, Solomon Yimer

**Affiliations:** 1 Department of Psychiatry, College of Medicine and Health Science, Dilla University, Dilla, Ethiopia; 2 School of Public Health, College of Medicine and Health Science, Dilla University, Dilla, Ethiopia; 3 Department of Midwifery, College of Medicine and Health Science, Dilla University, Dilla, Ethiopia

**Keywords:** Breakfast skipping, Children, Ethiopia, Sub-Saharan

## Abstract

Breakfast skipping and its relationship with academic achievement among primary school children were investigated in this study. A cross-sectional study was conducted among 848 primary school children. Breakfast skipping was analyzed using a 2-item questionnaire. A 19-item Social Academic and Emotional Behavior Risk Screening questionnaire was used to collect data on children’s behavior. The prevalence of breakfast skipping was found to be 38.1%. Living in a rural area (AOR = 5.2; 95% CI: 3.54, 7.71); having illiterate parents (AOR = 6.66; 95% CI 3.0, 14.7); having parents with a primary education level (AOR 5.18, 95% CI: 2.25, 11.94); living with guardians or other relatives (AOR = 4.06; 95%CI: 2.1, 7.9); and having lower academic achievement (AOR = 2.76; 95% CI: 1.44, 5.29) were factors associated with skipping breakfast.

In conclusion, breakfast skipping has been identified as a significant public health concern that requires an immediate response from stakeholders. It is recommended to intervene based on the identified factors.

## Introduction

Breakfast is frequently referred to as the “most important meal of the day.” However, there is little agreement on what constitutes breakfast. Researchers defined it as the “first meal of the day that breaks the fast after the longest length of sleep and is consumed within 2 to 3 hours of awaking”; and it can be eaten anywhere [1, 2]. Breakfast eating is thought to be a significant element in children’s cognitive health and academic achievement, but skipping breakfast is detrimental to both psychological and cognitive capabilities [1, 3].

Breakfast is suggested as part of a balanced diet because eating breakfast linked with the healthier macro and micronutrient intakes, BMI, and lifestyle [4]. Furthermore, children’s attendance and absenteeism may be connected to daily breakfast eating. However, in developing nations, the impacts of such supply on academic attainment remain unknown [5–9]. The World Health Organization recognizes that young people who develop healthy eating habits early in life are more likely to maintain maturity and to have reduced risk of chronic diseases [9, 10].

Schoolchildren in many underdeveloped nations face a variety of health and dietary issues. Furthermore, schools frequently lack basic utilities, all of which can have an impact on a child’s capacity to study [11]. Children who skipped breakfast were more likely to start drinking alcohol and smoking cigarettes, have bad behavioral and psychological difficulties, be absent from school frequently, and be less likely to exercise [8, 12, 13]. The percentage of people who skip breakfast has been reported to range between 1.7 and 30.0%. 39% of 13-year-olds skipped breakfast every school day, while the percentage rose to 45% among 15-year-olds. Furthermore, 2.5% of students skipped breakfast on all school days, while 14.0% skipped breakfast on at least one school day. The categories most likely to skip breakfast were those with a lower socioeconomic standing, non-whites, and females [14–16].

According to a few studies, 1.9% of students in selected government and private secondary schools in Addis Ababa never have breakfast during the week [17–21]. Another study from Ethiopia’s southern region found that 42.3% of people miss breakfast. The most common reasons for skipping breakfast were a loss of appetite, a lack of food, and a lack of time to eat [10]. Despite the fact that 41% of Ethiopians are under the age of 15, there is a paucity of information on breakfast skipping and its major contributing variables among schoolchildren. As a result, the purpose of this study was to look into the prevalence and correlates of breakfast skipping, as well as its relationship with academic performance, among school-aged children in Ethiopia’s Gedeo zone.

## Methods and materials

From February to March 2019, a cross-sectional institutional study was undertaken in Gedeo zone public primary schools. The Gedeo zone is located in Ethiopia’s South Region, 359 km from Addis Ababa. For the academic year 2018/2019 G.C., there were 225, 697 students registered in 241 elementary schools. During the data collecting period, the study sample consisted of school pupils aged 7 to 19 who were enrolled in a public primary school. Children who were gravely unwell, on the other hand, were not allowed to participate.

The sample size was calculated using a single population proportion formula with a margin of error of 5, 95% confidence interval, and a prevalence of breakfast skipping of 42.3% [10]. With a design effect of two and a 10% non-response rate, the sample size was 848. A multi-stage sampling procedure was used. The 241 schools were grouped together to form school clusters. Then, using a lottery system, 11 school clusters were chosen and the schools within the cluster considered as school stratum. Since schools was similar to each other in several aspects with the difference in the total number of students attending each stratum of schools, proportional allocation was used. The updated total number of students at each school strata and at each class (grade 1 to 8) was checked before the actual data collection period. The number of responders from the designated classes was determined via proportional allocation. Finally, using the list of students’ names as a sampling frame, study participants were chosen by lottery method.

Eleven teachers collected data under the supervision of four degree clinical nurses. A standardized questionnaire with socio-demographic, school bullying, Social, Academic, and Emotional Behavior Risk Screening (SAEBRS), and Parent/Guardian socio-demographic characteristic items was administered by the interviewer. Pre-testing of the questionnaire was done on 5% of the total sample size.

Breakfast skipping data was gathered using a two-item questionnaire that had been used in a prior study. Breakfast consumption on weekdays and weekend days was questioned about in the survey. The responses range from “never have breakfast” to “always have breakfast.” The overall score was divided into two categories: rarely having breakfast (never and 1-3 times per week) and frequently having breakfast (4 -7 times per week) [22].

The 2019 first term average score points from student roster reports were used to measure academic achievement. The student’s academic achievement was divided into two categories based on the data’s mean (69.08%): poor (scoring 69.08%) and good (score 69.08%). To measure students’ social, academic, and emotional conduct, the 19-item Social, Academic, and Emotional Behavior Risk Screening (SAEBRS) was employed. Subscales for Social, Academic, and Emotional Behavior are included in the instrument. With a dependability score of 0.93, the tool is suitable for elementary school [23, 24]. A total SAEBRS score of 0 to 36 is deemed “at risk,” whereas a score of 37 to 57 is regarded “not at danger.” [25].

Epi-data version 3.1 was used to code and enter the data, which was then exported to SPSS version 20 for analysis. Tables and graphs were used to summarize the data. We ran both bivariate and multivariate logistic regression analyses. In bivariate regression, we used the enter approach, and in multivariate regression, we used the Backward regression method. A *p*-value of less than 0.05 was considered statistically significant, and the strength of the link was determined using an adjusted odds ratio with a 95% confidence interval.

## Results

### Socio-demographic

A total of 848 school-aged children participated in the survey, with a response rate of 98%. Seventy percent (70.4%) of the children were between the ages of ten and fourteen. The average age of the participants was 12.2, with a standard deviation of 2.4 years. The ethnicity of the majority of the study participants was Gedeo (75.9%). More than 70 % of school-age children (*n* = 611, 72.1%) lived with both their fathers and mothers, with 555 (65.4%) living in rural areas. More over half of the parents of children (*n* = 480, 56.6%) were illiterate, and 454 (53.5%) were farmers (Table 1).

**Table 1:** Socio-demographic characteristics of public primary schools children in Gedeo Zone, Ethiopia, 2019 [*n* = 848]

Variable		Frequency(n)	Percent (%)
Age	5-9	106	12.5
	10-14	608	71.7
	15-19	134	15.8
Sex	Male	474	55.9
	Female	374	44.1
Religion	Orthodox Christian	169	19.9
	Muslims	32	3.8
	Protestant	626	73.8
	Catholic	21	2.5
Class Level study / Grade	Grade 1	101	11.9
	Grade 2	99	11.7
	Grade − 3	102	12.0
	Grade-4	96	11.3
	Grade-5	109	12.9
	Grade-6	112	13.2
	Grade-7	112	13.2
	Grade-8	117	13.8
Ethnic Background	Gedeo	644	75.9
	Oromo	65	7.7
	Amhara	60	7.1
	Sidama	41	4.8
	Others^a^	38	4.5
Respondents Living Arrangement	With both parents	611	72.1
	With mother or Father only	186	21.9
	With grandparents or other relatives	51	6.0
Children’s current address	Rural	555	65.4
	Urban	293	34.6
Family Educational Status father/mother	Unable to read and write	480	56.6
	Elementary School Complete	203	23.9
	Secondary School Complete	92	10.8
	Diploma and Above	73	8.6
Family occupation	Government Employee	84	9.9
	Merchant or/and other self-employee	164	19.3
	Farmer	454	53.5
	Others^b^	146	17.2

*NB* Others^a^ Wolaita, Gurage and Burji. Others^b^ daily laborers, jobless and house wife

### Breakfast consumption history

4.1% of study participants never ate breakfast throughout the week, 7.8% had one day per week, 12.8% had two days, 13.4% had three days, and 61.9% had four to seven days per week. Similarly, 4.7% of children reported never having breakfast on weekends, 14.6% reported having breakfast on only one weekend day, and 80.6% reported having breakfast on both weekend days. The prevalence of breakfast skipping was 38.1% (CI: 34.9, 41.3%).

### Social, academic and emotional behavior

**Fig. 1:**
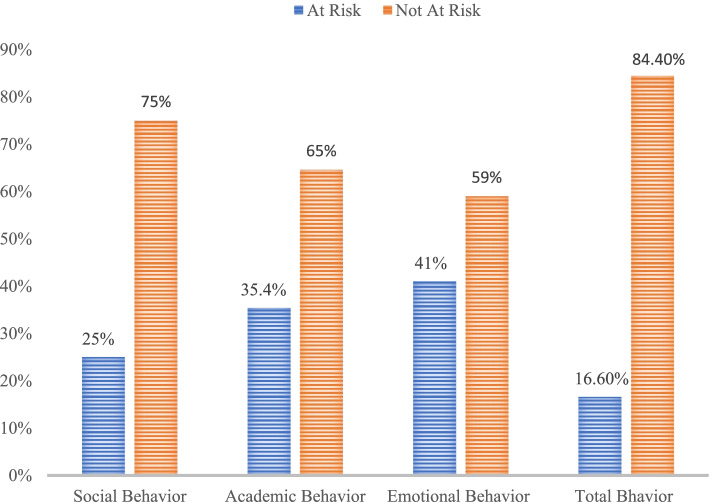
Social, academic, emotional behaviors of public primary schools children in Gedeo Zone, Ethiopia, 2019 [*n* = 848]. The data showed that two-fifth (*n* = 349) of children were a risk for emotional behavior problems; one fourth (*n* = 213) of them was a risk for social behavior problems; more than one third (*n* = 300) of them was a risk for academic behavior problems; and around one-sixth (*n* = 141) of them was a risk for social academic and emotional-behavioral problems

Regarding children behavior, two-fifth (*n* = 349, 41.1%) of them were a risk for emotional behavior problems; one fourth (*n* = 213, 25.1%) of them were a risk for social behavior problems; more than one third (*n* = 300, 35.4%) of them were a risk for academic behavior problems; and around one-sixth (*n* = 141, 16.6%) of them was a risk for all behavioral problems (Fig. 1).

### Factors associated with breakfast skipping

Breakfast skipping was linked to parents’ educational level, living in rural areas, living with one parent only; living with grandparents, and poor academic accomplishment, according to multivariable logistic regression.

Children with illiterate and primary education parents were 6 and 5 times more likely, respectively, to skip breakfast (AOR =6.65; 95% CI: 3.00, 14.74) and (AOR 5.18, 95% CI: 2.25, 11.94). Breakfast was more likely skipped by children who lived with grandparents or other relatives than by children who lived with both parents (AOR = 5.2; 95% CI: 3.54, 7.70).

Breakfast skipping was 5 times more likely among children who lived in rural areas (AOR = 4.96; 95% CI: 2.07, 7.79). Lower academic attainment was associated with a nearly threefold increase in the likelihood of skipping breakfast (AOR = 2.76; 95% CI: 1.44, 5.29). Furthermore, children who lived with only their father or mother were nearly 3.5 times (AOR =3.63; 95% CI: 2.40, 5.47) more likely than children who lived with both parents to skip breakfast (Table 2).

**Table 2:** Factors of Breakfast skipping among public primary schools children in Gedeo Zone, Ethiopia, 2019 [*n* = 848]

Variables		Breakfast Consumption		COR	AOR
		Often	Seldom		
Children Living circumstances	With Both Parents	437	174	1.00	1.00
	With mother or father only	65	121	4.675 (3.29, 6.63)	3.3 (2.15,5.19)**
	With grandparents or other relatives	23	28	3.05 (1.71, 5.45)	5.8 (2.8,11.8)**
Children’s’ current address	Rural	280	275	5.01 (3.52, 7.12)	4.7 (3.14, 7.21) **
	Urban	245	48	1.00	1.00
Children’s’ Family jobs	Government Employee	22	62	1.00	1.00
	Merchant or/and other self-employee	124	40	0.909(.49, 1.66)	0.69(.351.38)
	Farmer	261	193	2.08 (1.22,3.51)	1.4(.81, 2.6)
	Others^a^	78	68	2.45 (1.36, 4.41)	1.8 (.99,. 3.9)
Children’s’ Age group	5-9	80	26	1.00	1.00
	10-14.	369	239	1.99 (1.24, 3.19)	1.9 (0.7, 3.3)
	15-19	76	58	2.35 (1.34,4.11)	2.7(.99, 5.5)
Academic Behavior	At Risk	99	201	7.08 (5.18,9.7)	6.79 (4.6, 9.97)
	Not at Risk	426	122	1.00	1.00
Parent guardian Educational status	Illiterate	253	227	6.38 (3.1, 13.1)	5.3 (2.28, 12.36)*
	Elementary School Complete	134	69	3.66 (1.72,7.79)	4.02 (1.65, 9.8) *
	Secondary School Complete	74	18	1.7(.72, 4.12)	1.06(.37, 2.9)
	Diploma and Above	64	9	1.00	1.00
Children’s Academic achievement	Poor to Fair (less than 50 to 59%)	244	229	2.8 (2.083.77)	2.57(1.77, 3.7) *
	Satisfactory to Excellent (60-79%, 80-89% & 90 - 100%)	281	94	1.00	1.00

*Note* * = *P* < 0.001; ** *P* < 0.001; COR- crude odds ratio AOR-Adjusted odds ratio 1: reference category; others^a^: daily laborers, house wife and jobless

## Discussion

The prevalence of breakfast skipping was found to be 38.1% in the current study (CI: 34.9 to 41.3%). The result of the current study is in line with other study [26]. This consistency may be due to sharing of the same study population with the same age group was used, as well as the use of the same assessment method. According to certain school-based study findings, the general rate of breakfast skipping is between 10 and 66% [8, 27–34]. But, this finding is much higher than study results in America (10 - 30%) [35], Saud Arabia (19.6%) [36], Iran (18 - 26%) [37], Brazil (7%) [38] and Canada (10%) [39]. Differences in socio-cultural practice, dietary preferences, and the concept of breakfast intake could be the cause of the disparity. On the other hand, the findings are consistent with Chinese research (38.7%) [40], Dutch (38%) [41] as well as the Canadian Child Hunger Survey (42%) [42]. While this study is lower than in Sidama, Ethiopia and Rives state, Nigeria [43, 44].

Children living with illiterate parents or guardians were more than six times more likely to skip breakfast than children living with well-educated parents or guardians (AOR = 6.66, 95%) CI: 3.0, 14.75). Similarly, children with elementary school completing parents/guardians were 5 times more likely to skip breakfast (AOR = 5.18, 95% CI 2.25, 11.9). Different studies also reported a similar findings [2, 37, 43, 45–47]. The possible justification for this is that illiterate parents are less likely to understand the cognitive and physical benefits of regular breakfast consumption for their children. That is, parent education programs have the potential to alter the eating behaviors that young children develop and increase the healthy behaviors they engage in throughout life. Besides studies reported that education might have more impact on the nutritional status of the next generation if school curricula focused on directly improving health and nutritional knowledge of parents [48]. Though previous studies recommend a possible interventions thorough provision of free *breakfast* at *schools* and regular education for parents about healthy dieting in children to decrease *breakfast skipping* [49–51]*.*

Rural children were 5 times more likely than urban children to skip breakfast (AOR = 5.20, 95% CI 3.54, 7.71). An increased level of breakfast skipping in the rural communities is shown, particularly in Ethiopia, because the rural populations have adopted a breakfast-free lifestyle due to food insecurity and food shortages [52]. This finding is consistent with the findings of other studies [53]. The other justification for the rural prevalence of breakfast declining faster than urban is probably because rural students have more stress, such as fear of failing and financial issues [54].

Furthermore, children who lived with a father or mother were only 3.5 times (AOR =3.63; 95% CI: 2.40, 5.47) more likely than their peers to skip breakfast. Similarly, children who lived with grandparents or other relatives were four times (AOR = 4.06, 95% CI 2.07, 7.97) more likely than children who lived with both parents to skip breakfast. It’s possible that relatives other than primary parents were less worried about children, particularly in Ethiopia. Single parents, on the other hand, may be financially strapped and unable to provide a consistent breakfast for their children. Similar results have been reported [2, 34, 37, 45].

Finally, low academic achievers were three times more likely than higher achievers to forgo breakfast. Other investigations have come to similar conclusions [8, 10, 16].. This can be explained by the apparent benefits of having daily breakfast consumption on increasing school attendance and reducing absenteeism [5–9].

## Conclusions and future directions

Breakfast skipping was discovered to be relatively common in this study. Breakfast consumption has been linked to poor academic performance. Parental illiteracy and primary education, children’s address, children living with only their mother or father, children living with grandparents, and low academic achievement were all significant predictors.

As a result, it is preferable to implement and work on children’s and education acts by raising awareness about the benefits of daily breakfast for children, especially among parents in rural areas, and cooperating to support children with low academic standing, living with a single parent, and without primary parents. It is also critical to implement school health and nutrition initiatives and collaborate with key stakeholders such as child care providers and other professionals who work with young children and their families to improve children’s eating habits. Furthermore, it is also suggested that future research address the effect of breakfast consumption on children’s academic and cognitive performance through a follow-up study.

## Limitations

The current study was limited to assessing a lack of food and a lack of time to eat, both of which could be modifiable determinants in the causative pathway of breakfast skipping. Another limitation of this study is that, due to the cross-sectional nature of the study design, it does not show any cause-effect relationship. Furthermore, the nutritional state of the children and their learning difficulties were not assessed in this study.

## Data Availability

This published article contains all of the data generated or analyzed during this investigation. The current study’s data sets are available upon reasonable request from [Lulu Abebe, email: luluasayo1154@gmail.com; Mobile: + 251970975865, Dilla university, Dilla and the corresponding author].

## Abbreviations

AOR: Adjusted odds ratio
BMI: Body Mass Index
IRB: Institutional Review Board (IRB)
CI: Confidence interval
SNNPR: South Nation’s Nationality Peoples Region

## References

[1] O'Neil CE, Byrd-Bredbenner C, Hayes D, Jana L, Klinger SE, Stephenson-Martin S (2014). The role of breakfast in health: definition and criteria for a quality breakfast. J Acad Nutr Diet.

[2] Pearson N, Biddle SJ, Gorely T (2009). Family correlates of breakfast consumption among children and adolescents. A systematic review. Appetite.

[3] Oddy WH, Robinson M, Ambrosini GL, Therese A, de Klerk NH, Beilin LJ (2009). The association between dietary patterns and mental health in early adolescence. Prev Med.

[4] Affinita A, Catalani L, Cecchetto G, De Lorenzo G, Dilillo D, Donegani G (2013). Breakfast: a multidisciplinary approach. Ital J Pediatr.

[5] Kleinman RE, Hall S, Green H, Korzec-Ramirez D, Patton K, Pagano ME (2002). Diet, breakfast, and academic performance in children. Ann Nutr Metab.

[6] Widenhorn-Müller K, Hille K, Klenk J, Weiland U (2008). Influence of having breakfast on cognitive performance and mood in 13-to 20-year-old high school students: results of a crossover trial. Pediatrics.

[7] Hoyland A, Dye L, Lawton CL (2009). A systematic review of the effect of breakfast on the cognitive performance of children and adolescents. Nutr Res Rev.

[8] Rampersaud GC, Pereira MA, Girard BL, Adams J, Metzl JD (2005). Breakfast habits, nutritional status, body weight, and academic performance in children and adolescents. J Am Diet Assoc.

[9] Kearns R, Ameratunga S, Neuwelt P. Widening the lens on child health. N Z Med J. 2005;118(1227). 16372034

[10] Adole AA, Singh P, Bosha T, Desalegn BB (2015). Effect of breakfast eating patterns and anthropometric measurements on cognitive function of early adolescents in rural area of Sidama zone, southern Ethiopia. J Food Nutr Sci.

[11] Powell CA, Walker SP, Chang SM, Grantham-McGregor SM (1998). Nutrition and education: a randomized trial of the effects of breakfast in rural primary school children. Am J Clin Nutr.

[12] Keski-Rahkonen A, Kaprio J, Rissanen A, Virkkunen M, Rose RJ (2003). Breakfast skipping and health-compromising behaviors in adolescents and adults. Eur J Clin Nutr.

[13] Kar BR, Rao SL, Chandramouli B (2008). Cognitive development in children with chronic protein energy malnutrition. Behav Brain Funct.

[14] Gibney MJ, Barr SI, Bellisle F, Drewnowski A, Fagt S, Livingstone B (2018). Breakfast in human nutrition: the international breakfast research initiative. Nutrients.

[15] Mullan B, Singh M (2010). A systematic review of the quality, content, and context of breakfast consumption. Nutr Food Sci..

[16] Boschloo A, Ouwehand C, Dekker S, Lee N, De Groot R, Krabbendam L (2012). The relation between breakfast skipping and school performance in adolescents. Mind Brain Educ.

[17] Roba K, Abdo M, Wakayo T (2016). Nutritional status and its associated factors among school adolescent girls in Adama City, Central Ethiopia. J Nutr Food Sci.

[18] Gebreyohannes Y, Shiferaw S, Demtsu B, Bugssa G (2014). Nutritional status of adolescents in selected government and private secondary schools of Addis Ababa, Ethiopia. Adolescence.

[19] Jebena MG, Lindstrom D, Belachew T, Hadley C, Lachat C, Verstraeten R (2016). Food insecurity and common mental disorders among Ethiopian youth: structural equation modeling. PLoS One.

[20] Mulugeta A, Hagos F, Stoecker B, Kruseman G, Linderhof V, Abraha Z (2009). Nutritional status of adolescent girls from rural communities of Tigray, northern Ethiopia. Ethiop J Health Dev.

[21] Hadley C, Tegegn A, Tessema F, Cowan JA, Asefa M, Galea S (2008). Food insecurity, stressful life events and symptoms of anxiety and depression in East Africa: evidence from the Gilgel gibe growth and development study. J Epidemiol Community Health.

[22] Lien L (2007). Is breakfast consumption related to mental distress and academic performance in adolescents?. Public Health Nutr.

[23] Gresham FM, Elliott SN, Vance MJ, Cook CR. Comparability of the social skills rating system to the social skills improvement system: content and psychometric comparisons across elementary and secondary age levels. Sch Psychol Q. 2011;26(1):27–44.

[24] Kamphaus RW, Reynolds CR (2007). BASC-2 Behavioral and Emotional Screening System manual.

[25] Kettler RJ, Elliott SN, Davies M, Griffin P. Testing a multi-stage screening system: predicting performance on Australia's national achievement test using teachers' ratings of academic and social behaviors. Sch Psychol Int. 2012;33:93–111.

[26] Hopkins LC, Sattler M, Steeves EA, Jones-Smith JC, Gittelsohn J (2017). Breakfast consumption frequency and its relationships to overall diet quality, using healthy eating index 2010, and body mass index among adolescents in a low-income urban setting. Ecol Food Nutr.

[27] Thompson-McCormick JJ, Thomas JJ, Bainivualiku A, Khan AN, Becker AE (2010). Breakfast skipping as a risk correlate of overweight and obesity in school-going ethnic Fijian adolescent girls. Asia Pac J Clin Nutr.

[28] Al Turki M, Al Shloi S, Al Harbi A, Al Agil A, Philip W, Qureshi S (2018). Breakfast consumption habits among schoolchildren: a cross-sectional study in Riyadh, Saudi Arabia. Int Res J Med Med Sci.

[29] Acham H, Kikafunda J, Malde M, Oldewage-Theron W, Egal A (2012). Breakfast, midday meals and academic achievement in rural primary schools in Uganda: implications for education and school health policy. Food Nutr Res.

[30] Alexy U, Wicher M, Kersting M (2010). Breakfast trends in children and adolescents: frequency and quality. Public Health Nutr.

[31] Bin Zaal A, Musaiger A, D'Souza R (2009). Dietary habits associated with obesity among adolescents in Dubai. United Arab Emirates Nutr Hospit.

[32] Kawafheh MM, Hamdan FR, Abozeid SE-S, Nawafleh H (2014). The effect of health education programs for parents about breakfast on students' breakfast and their academic achievement in the north of Jordan. Int J Advanc Nurs Stud.

[33] Kawafha M (2013). Impact of skipping breakfast on various educational and overall academic achievements of primary school children in northern of Jordan. Aust J Basic Appl Sci.

[34] Vereecken C, Dupuy M, Rasmussen M, Kelly C, Nansel TR, Al Sabbah H (2009). Breakfast consumption and its socio-demographic and lifestyle correlates in schoolchildren in 41 countries participating in the HBSC study. Int J Public Health.

[35] Sampson AE, Dixit S, Meyers AF, Houser R (1995). The nutritional impact of breakfast consumption on the diets of inner-city African-American elementary school children. J Natl Med Assoc.

[36] ALBashtawy M (2017). Breakfast eating habits among schoolchildren. J Pediatr Nurs.

[37] Ghafari M, Doosti-Irani A, Amiri M, Cheraghi Z (2017). Prevalence of the skipping breakfast among the Iranian students: a review article. Iran J Public Health.

[38] Pereira JL, de Castro MA, Hopkins S, Gugger C, Fisberg RM, Fisberg M (2018). Prevalence of consumption and nutritional content of breakfast meal among adolescents from the Brazilian National Dietary Survey. J Pediatr (Versão em Português).

[39] Barr SI, DiFrancesco L, Fulgoni VL (2014). Breakfast consumption is positively associated with nutrient adequacy in Canadian children and adolescents. Br J Nutr.

[40] Wang M, Zhong J-M, Wang H, Zhao M, Gong W-W, Pan J (2016). Breakfast consumption and its associations with health-related behaviors among school-aged adolescents: a cross-sectional study in Zhejiang Province, China. Int J Environ Res Public Health.

[41] Croezen S, Visscher T, Ter Bogt N, Veling M, Haveman-Nies A (2009). Skipping breakfast, alcohol consumption and physical inactivity as risk factors for overweight and obesity in adolescents: results of the E-MOVO project. Eur J Clin Nutr.

[42] Basrur S (1998). Child nutrition programs in Toronto. Report to the Toronto board of Health.

[43] Adole AA, Ware MB (2014). Assessment of breakfast eating habits and its association with cognitive performance of early adolescents (11-13 years) in Shebedino District, Sidama zone, southern Ethiopia. J Food Nutr Sci.

[44] Owo WJ, Nwala L (2019). Impacts of consumption of meal on academic achievement in basic science among junior secondary students in Rivers state. KIU J Soc Sci.

[45] Øverby N, Stea TH, Vik FN, Klepp K-I, Bere E (2011). Changes in meal pattern among Norwegian children from 2001 to 2008. Public Health Nutr.

[46] Arnold DH, Doctoroff GL (2003). The early education of socioeconomically disadvantaged children. Annu Rev Psychol.

[47] Bradley RH, Corwyn RF (2002). Socioeconomic status and child development. Annu Rev Psychol.

[48] Alderman H, Headey DD (2017). How important is parental education for child nutrition?. World Dev.

[49] Larson N, Wang Q, Grannon K, Wei S, Nanney MS, Caspi C (2018). A low-cost, grab-and-go breakfast intervention for rural high school students: changes in school breakfast program participation among at-risk students in Minnesota. J Nutr Educ Behav.

[50] Christensen CB, Mikkelsen BE, Toft U (2019). The effect of introducing a free breakfast club on eating habits among students at vocational schools. BMC Public Health.

[51] Kesztyüs D, Traub M, Lauer R, Kesztyüs T, Steinacker JM (2017). Skipping breakfast is detrimental for primary school children: cross-sectional analysis of determinants for targeted prevention. BMC Public Health.

[52] Mohamed AA (2017). Food security situation in Ethiopia: a review study. Int J Health Econ Policy.

[53] Liu J, Hwang W-T, Dickerman B, Compher C (2013). Regular breakfast consumption is associated with increased IQ in kindergarten children. Early Hum Dev.

[54] Ba T, Liu Z, Guo W, Eshita Y, Sun J (2013). Comparison of breakfast consumption in rural and urban among Inner Mongolia Medical University students.

